# *Plasmodium* Infection–Cure Cycles Increase the Capacity of Phagocytosis in Conventional Dendritic Cells

**DOI:** 10.3390/pathogens12101262

**Published:** 2023-10-19

**Authors:** Ryosuke Adachi, Takahiko Tamura

**Affiliations:** School of Pharmacy, Kanazawa University, Kakuma-machi, Kanazawa 920-1192, Japan

**Keywords:** malaria, dendritic cells, phagocytosis

## Abstract

Malaria stands as one of the most pervasive human infectious diseases globally and represents a prominent cause of mortality. Immunity against clinical malaria disease is achieved through multiple infection and treatment cycles, culminating in a substantial reduction in parasite burden. To investigate this phenomenon, we established a murine model involving repeated infection–cure cycles, whereby mice were infected with the lethal rodent malarial parasite *Plasmodium berghei* ANKA and subsequently treated with the anti-malarial drug pyrimethamine. Our earlier study revealed a significant decrease in the capacity of conventional dendritic cells (cDCs) to produce cytokines upon stimulation in infection-cured mice. In the present study, we aimed to further elucidate the modulation of cDC functionality during repeated infection–cure cycles by examining their phagocytic capacity. Administering fluorescent beads to mice resulted in no significant difference in the total number of bead-positive cells within the spleens of both uninfected and 3-cure (three cycles of infection–cure) mice. However, the proportion of the CD11c^+^F4/80^−^ population within bead-positive cells was notably higher in 3-cure mice compared to uninfected mice. Subsequent in vitro analysis of bead phagocytosis by purified CD11c^+^cDCs revealed that the cDC2 subset from 3-cure mice exhibited significantly enhanced phagocytic capacity in comparison to their uninfected counterparts. These findings underscore the substantial impact of repeated infection–cure cycles on various facets of cDC function, potentially influencing the trajectory of immune responses against subsequent malaria infections.

## 1. Introduction

Malaria, a global affliction caused by *Plasmodium* parasites, represents one of the world’s potentially fatal diseases [1]. Infection with *Plasmodium falciparum*, the primary parasitic agent responsible for this malady in humans, engenders a broad spectrum of pathologies, with cerebral malaria (CM) being the most lethal manifestation. Unlike numerous other infectious diseases, humans do not readily develop robust immunological memory against malarial parasites, thus presenting a formidable obstacle to malaria eradication. Nonetheless, it is well documented that in regions with high malaria transmission rates, adults exposed repeatedly to these parasites acquire immunity, rendering them less susceptible to severe symptoms such as high parasitemia and CM [2]. Nevertheless, the precise mechanism underpinning the acquisition of immunity and the development of immunological memory remains elusive. Understanding these mechanisms may pave the way for the development of more effective strategies to resist malaria parasite infections, including the progress of malaria vaccines.

CD11c^+^ dendritic cells (DCs) play pivotal roles in initiating both innate and adaptive immunity during microbial infections, primarily through Toll-like receptors (TLRs), cytosolic nucleic acid sensors, and inflammasomes, among other mechanisms [3]. It is widely acknowledged that the spleen constitutes a critical site for the induction of anti-malaria immunity [4]. Murine splenic DCs can be classified into two main categories: conventional DCs (cDCs) and plasmacytoid DCs (pDCs). Among these, cDCs stand out as the most potent antigen-presenting cells, as they efficiently capture antigens and subsequently present them to naive T cells, thereby facilitating their functional differentiation into effector T cells. Additionally, cDCs secrete a diverse array of cytokines, including IL-12 and TNF, which play pivotal roles in orchestrating immune responses against antigens. Further stratification of murine splenic cDCs reveals two distinct subsets: Th1-biased cDC1 (CD8^+^CD11b^−^) and Th2-biased cDC2 (CD8^−^CD11b^+^), primarily distinguished by their surface molecule expression profiles [5].

The roles of cDC1 and cDC2 subsets in anti-malaria immunity have yet to be fully elucidated. Previous studies have demonstrated that cDC1 possesses cross-priming functions and is superior to cDC2 in activating antigen-specific CD8^+^ T cells [6]. While activated CD8^+^ T cells play a role in experimental cerebral malaria (ECM), their significance in eliminating blood-stage malaria parasites is limited. Recent studies have shown that the cDC1 subset is importantly involved in the activation of CD4^+^ T cells, in addition to CD8^+^ T cells, during malaria infection [7]. Thus, the central role of cDCs in initiating immunity against the primary infection of malaria parasites has been recently unveiled. However, their role in establishing immunological memory and resulting protection against malaria reinfection remains largely unexplored.

Phagocytosis is the biological process of engulfing particles larger than 0.5 μm [8]. This crucial process plays an essential role in initiating immune responses against pathogenic microbes by facilitating their elimination within phagolysosomes, which are formed through the fusion of phagosomes and lysosomes. Additionally, phagocytosis enables the presentation of digested antigens to T cells. Among the professional phagocytes, which include macrophages and granulocytes, cDCs are known to possess the ability to phagocytose pathogenic microbes, including *Plasmodium*-infected erythrocytes [9].

To investigate alterations in the characteristics of cDCs and their roles following repeated infection–cure treatments, we previously established a mouse model involving cycles of *Plasmodium berghei* ANKA (PbA) infection and subsequent cure. The three cycles of infection and cure in this model (called 3-cure mice) demonstrated a potent anti-malarial effect, including the inhibition of parasitemia growth and the prevention of ECM after PbA infection challenge [10]. Examination of splenic cDCs indicated that PbA infection–cure treatment induced trained immunity in cDCs, resulting in the modulation of cytokine production upon Toll-like receptor stimulation. This modulation was sustained for a long term (6 months).

These findings prompted us to undertake a more comprehensive analysis of cDCs in infection-cured mice, with the potential to contribute to the development of more effective strategies for resisting reinfection with malaria parasites as well as more efficient immune responses against reinfection with malaria parasites. The alterations in the phagocytic capabilities of cDCs may also hold implications for immunity against other microbial infections, in addition to the erythrocytic stage of malaria parasites. In this study, we conducted an analysis of cDCs phagocytosis activity using fluorescent beads. Our hypothesis posited that the modulation of phagocytosis activity in cDCs might be involved in the immune responses observed in 3-cure mice. The results demonstrated that cDCs from infection-cured mice exhibited increased phagocytosis activity compared to those from uninfected mice.

## 2. Materials and Methods

### 2.1. Ethics Statement

All animals were housed and cared for in the institutional animal facility at Kanazawa University. The animal experiments presented in this study were conducted in strict compliance with the guidelines provided by the Institutional Animal Care Committee of Kanazawa University (Approval No. 24098-1).

### 2.2. Mice and Parasite Infection

C57BL/6 (B6) female mice were purchased from SLC (Hamamatsu, Japan). Lethal rodent malaria parasite PbA, used in this study, has been described previously [11]. For infection, mice were intraperitoneally (i.p.) inoculated with 2 × 10^4^ parasitized red blood cells (pRBCs). The levels of parasitemia in the infected mice were monitored by microscopic examination of Giemsa-stained tail blood smears. The infection–cure cycle treatment was repeated, and 3-cure mice were established as previously described [10]. The infection-cured mice were used for further experiments 3 weeks after last treatment.

### 2.3. PCR

The blood from uninfected, 3-cure, or PbA-infected (3% parasitemia) mice were collected. The genomic DNA from blood were purified by QIAamp DNA blood kit (QIAGEN, Hilden, Germany). The concentration of DNA was measured using Nanodrop2000 spectrophotometer (Thermo Fisher Scientific, Waltham, MA, USA). The indicated amounts of DNA of each sample were used for PCR. The primer sequences (derived from PbMSP1 XM_034564492.1) used for PCR were 5′- GCTACTACCGATAAAGATGAAAAAAAG -3′ and 5′- TAATAAATCCATGCTATCCATATTAAGCAT-3′. For the amplification of target sequences, standard PCR reagents and protocol were used. The temperature profile included 94 °C for 30 s and 35 cycles of denaturation at 94 °C for 30 s, annealing at 53 °C for 30 s, and extension at 72 °C for 20 s. The PCR products were analyzed by standard agarose gel electrophoresis.

### 2.4. Flow Cytometric Analysis

The procedure of flow cytometric analysis of splenocytes was previously described [11]. Splenocytes were preincubated with anti-CD16/32 monoclonal antibody (mAb) to block Fc receptor and avoid non-specific binding of antibodies of interest to cells and stained with fluorochrome-conjugated mAbs. The antibodies used included PE-anti-CD11c (N418), FITC or PE-Cy7-anti-CD8a(53–6.7) and FITC-anti-TCRb (H57-597), FITC-anti-F/40(BM8), PE-Cy7-anti-CD11b(M1/70), PE-Cy7-anti-B220(RA3-6B2), PE-Cy7-anti-Gr-1(RB6-8C5), PE-Cy7-anti-CD19(6D5), PE-Cy7-anti-CD3e(145-2C11), APC-Cy7-anti-MHC class II (M5/114.15.2) mAbs. All antibodies used in this study were purchased from BioLegend (San Diego, CA, USA). Data acquisition was carried out using a FACSVerse^TM^ flow cytometer and subsequent analysis was performed using the FACSuite^TM^ software version 1.0.6 (BD Biosciences, San Jose, CA, USA). All datasets were analyzed after gating leukocytes with forward-scatter/side-scatter gates. Dead cells with low forward-scatter were gated out.

### 2.5. In Vivo Phagocytosis Assay

For the study of phagocytic ability in vivo, mice were inoculated intravenously (i.v.) with polystyrene crimson fluorescent beads (1 μm, Molecular Probes, Eugene, OR, USA, 1.6 × 10^8^ microspheres/100 μL PBS) according to previously described procedure [12]. After 2 h, splenocytes were stained with FITC-anti-F4/80, PE-anti-CD11c, PE-Cy7-anti-CD11b, PE-Cy7-anti-CD3e, PE-Cy7-anti-CD19, PE-Cy7-anti-Gr-1, anti-CD16/32. The stained cells were analyzed using flow cytometer. The fluorescence of crimson beads was detected in APC channel.

### 2.6. In Vitro Phagocytosis Assay

For purification of CD11c^+^ cDCs from the spleens, splenocytes from mice were incubated with biotinylated anti-CD11c (N418; Biolegend), and CD11c^+^ cDCs were collected using streptavidin particles (BD Biosciences) and cell separation magnet (BD Biosciences).

For the study of phagocytic ability in vitro [12], splenic DCs (1 × 10^5^ cells per well) from uninfected and 3-cure mice were seeded on 24-well plates and cultured in the presence of crimson fluorescent beads (1 × 10^7^ microspheres) in 1 mL of culture medium (RPMI1640 medium supplemented with 10% fetal bovine serum, antibiotics, sodium pyruvate, L-glutamine, and 2-mercaptoethanol) at 37 or 4 °C for 2 h. After incubation, the cells were kept on ice. The cells were collected and stained with FITC-anti-CD8a, PE-anti-CD11c, PE-Cy7-anti-CD11b, anti-CD16/32, and were analyzed using flow cytometer as described above. 

### 2.7. Statistical Analysis

Results were presented as mean ± SE. Statistical analysis was conducted using the Mann–Whitney U test to compare two experimental groups, and data calculations were carried out using GraphPad Prism version 5 (GraphPad Software, San Diego, CA, USA). A *p*-value of less than 0.05 was deemed statistically significant. 

## 3. Results

### 3.1. PCR Analysis of Blood Samples from 3-Cure Mice

In this study, PbA parasites were initially introduced into mice, followed by treatment with an anti-malarial drug. The presence or absence of parasites in the blood samples was assessed using the smear method, which involves microscopic analysis of Giemsa-stained blood films. This method confirmed the absence of parasites in the blood films, with a count of 1000 cells, indicating successful treatment. However, it is important to note that the smear method may potentially overlook trace amounts of parasites in cured mice due to its lower sensitivity. Therefore, to ensure the complete absence of malaria parasites, we employed PCR analysis, a more sensitive technique compared to the smear method [13].

**Figure 1:**
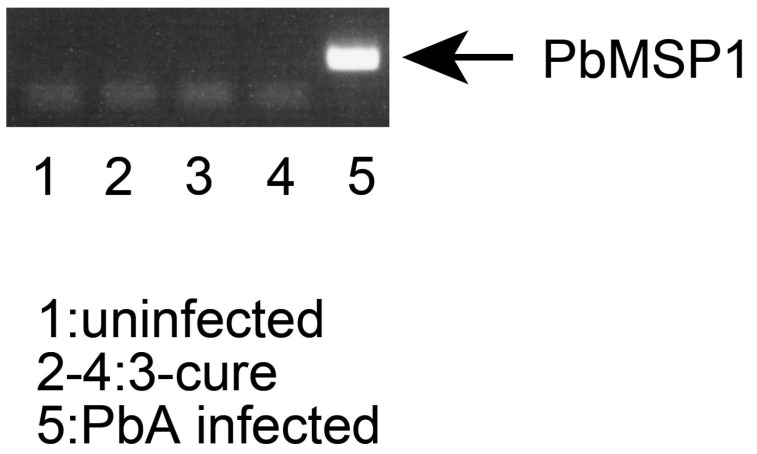
PCR check of blood DNA from 3-cure mice. The genomic DNA was purified from the blood of mice. The DNA (5 ng for uninfected and PbA-infected mice, 25 ng for each from three 3-cure mice) was used as templates of amplification of PbMSP1 gene. The PCR products (345 bp) were analyzed by agarose (2%) gel electrophoresis. Arrow indicates the amplification of PbMSP1 target. lane1: uninfected. lane2–4: 3-cure. lane5: PbA infected.

Blood samples were collected from three groups: uninfected mice, 3-cure mice (those treated with anti-malarial drugs), and PbA-infected mice (infected with PbA parasites but without anti-malarial drug treatment). The DNA from these blood samples was isolated and subjected to PCR analysis. As shown in Figure 1, while PCR products from the PbMSP1 gene were readily detected in the blood samples from PbA-infected mice, no PCR products were detected in the samples from 3-cure mice, even when a larger amount of DNA sample was used as a PCR template. Similar results were obtained when PCR was conducted using the Pb18S rRNA gene. These findings provide robust evidence that PbA parasites were effectively eliminated from infected mice through anti-malarial treatment, confirming the suitability of these mice for further experiments in this study.

### 3.2. The Phagocytosis Assay In Vivo

**Figure 2:**
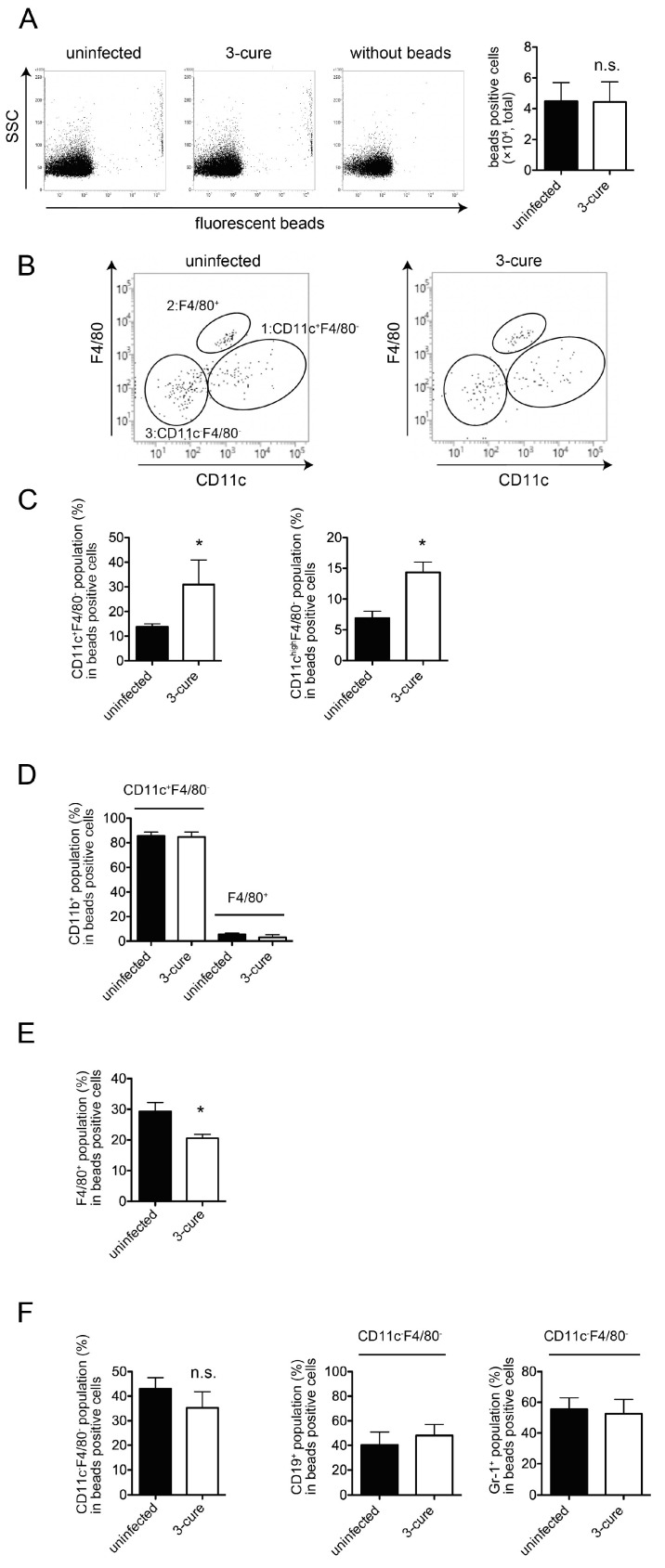
The percentage of CD11c^+^F4/80^−^ cDCs population was significantly increased in 3-cure mice in in vivo phagocytosis assay. Uninfected or 3-cure mice were inoculated i.v. with fluorescent beads. Two hours later, splenocytes were analyzed using flow cytometry. (**A**) The representative flow cytometric plots are shown. The total cell number of fluorescent-positive cells (n = 3 for each group) are shown as mean ± SE. (**B**) Splenocytes were stained for F4/80 and CD11c, and the representative plots are shown after gating for fluorescent-positive cells. (**C**) The percentage of CD11c^+^F4/80^−^ cells (n = 3 for each group) or CD11c^high^F4/80^−^ cells (n = 5 for each group) within fluorescent-positive cells. (**D**) The expression of CD11b of CD11c^+^F4/80^−^ and F4/80^+^ population within bead-positive cells (n = 6 for each group). (**E**) The percentage of F4/80^+^ within fluorescent-positive cells (n = 4 for each group). (**F**) CD11c^−^F4/80^−^ cells within fluorescent-positive cells (n = 4 for each group). The percentage of CD19 or Gr-1 positive cells was also demonstrated (n = 3 for each group). The experiments were performed four times and representative data are shown. Asterisks denote that a significant difference was statistically observed between uninfected and 3-cure mice (*p* < 0.05). n.s means not significant difference between uninfected and 3-cure mice.

In the in vivo phagocytosis assay, we compared the phagocytic capacity of 3-cure mice with that of age-matched uninfected mice by i.v. administering fluorescent beads into both groups. Subsequently, the mice were sacrificed, and splenocytes were prepared for the analysis of bead-positive cells using flow cytometry. As illustrated in Figure 2A, the total number of bead-positive cells in 3-cure mice was not significantly different from that in uninfected mice. This indicates that the 3-cure cycle treatment did not enhance the capacity for in vivo phagocytosis within the spleen itself. 

CD11c^+^ cDCs as well as F4/80^+^ macrophages would be included in bead-positive phagocytes in the spleens [12]. Further examination of the bead-positive cells based on the expression of CD11c and F4/80 revealed three main populations: (1) CD11c^+^F4/80^−^, (2) F4/80^+^, and (3) CD11c^−^F4/80^−^ (Figure 2B). The CD11c^+^F4/80^−^ population was found to be CD3e^−^class II^+^, suggesting that this population contained cDCs. Notably, in 3-cure mice, the percentage of CD11c^+^F4/80^−^ cells within bead-positive cells was significantly increased compared to uninfected mice, although the difference was not so drastic (Figure 2C). Moreover, the percentage of the CD11c^high^ F4/80^−^ population within bead-positive cells in 3-cure mice was also significantly elevated compared to uninfected mice (Figure 2C).

In a previous study, it was demonstrated that PbA infection led to a reduction in the number of cDCs [14]. However, in this study, the total cell number of splenic cDCs in 3-cure mice did not differ significantly from that in uninfected mice, while the percentage of CD8^+^CD11b^−^ (cDC1 subset) within the cDC population was significantly decreased in 3-cure mice compared to uninfected mice as previously described [10]. However, the percentage of CD11b positivity within bead-positive CD11c^+^F4/80^−^ cells was not different between uninfected and 3-cure mice (Figure 2D). 

The F4/80^+^ population was CD11b^−^ (Figure 2D), and these cells were consistent with red pulp macrophages [12]. A previous study demonstrated that PbA infection reduced F4/80^+^ splenic red pulp macrophages [12]. However, in this study, 3-cure mice significantly restored the total cell number of splenic F4/80^+^ red pulp macrophages, which was not significantly different from that in uninfected mice. Interestingly, the percentage of F4/80^+^ cells within bead-positive cells in 3-cure mice was significantly lower than that in uninfected mice (Figure 2E), in contrast to the CD11c^+^F4/80^−^ population. While the percentage of F4/80^+^ cells within bead-positive cells was higher than that of CD11c^+^F4/80^−^ cells in uninfected mice, the situation was reversed in 3-cure mice.

The percentage of CD11c^−^F4/80^−^ cells within bead-positive cells in 3-cure mice did not change significantly compared to that in uninfected mice (Figure 2F). The previous studies demonstrate a part of B cells possess phagocytosis ability [15]. Monocytes and granulocytes phagocytose *Plasmodium*-infected RBCs [16,17]. Actually, the bead-positive CD11c^−^F4/80^−^ population was CD19^+^ or Gr-1^+^ (Figure 2F), but CD3e^−^, suggesting that this population contained a mixture of B cells and Gr-1^+^ cells (monocytes and granulocytes).

In summary, repeated infection–cure cycles increased the percentage of cDCs within the total bead-positive cell population, indicating that blood-derived materials were more likely to be incorporated into cDCs in the spleen.

### 3.3. The Phagocytosis Assay In Vitro

One possible explanation for the in vivo phagocytosis results was that the capacity for phagocytosis of cDCs might be increased in 3-cure mice. To investigate the capacity for phagocytosis of cDCs in vitro, we purified total CD11c^+^ cDCs from the spleens of both uninfected mice and 3-cure mice. The purified CD11c^+^ cDCs used in this study expressed MHC class II molecules and included both cDC1 and cDC2 subsets, as well as unpurified cDCs. We introduced fluorescent beads to the CD11c^+^ cDCs and analyzed them using flow cytometry, distinguishing between cDC1 and cDC2 subsets based on the expressions of CD8 and CD11b.

**Figure 3:**
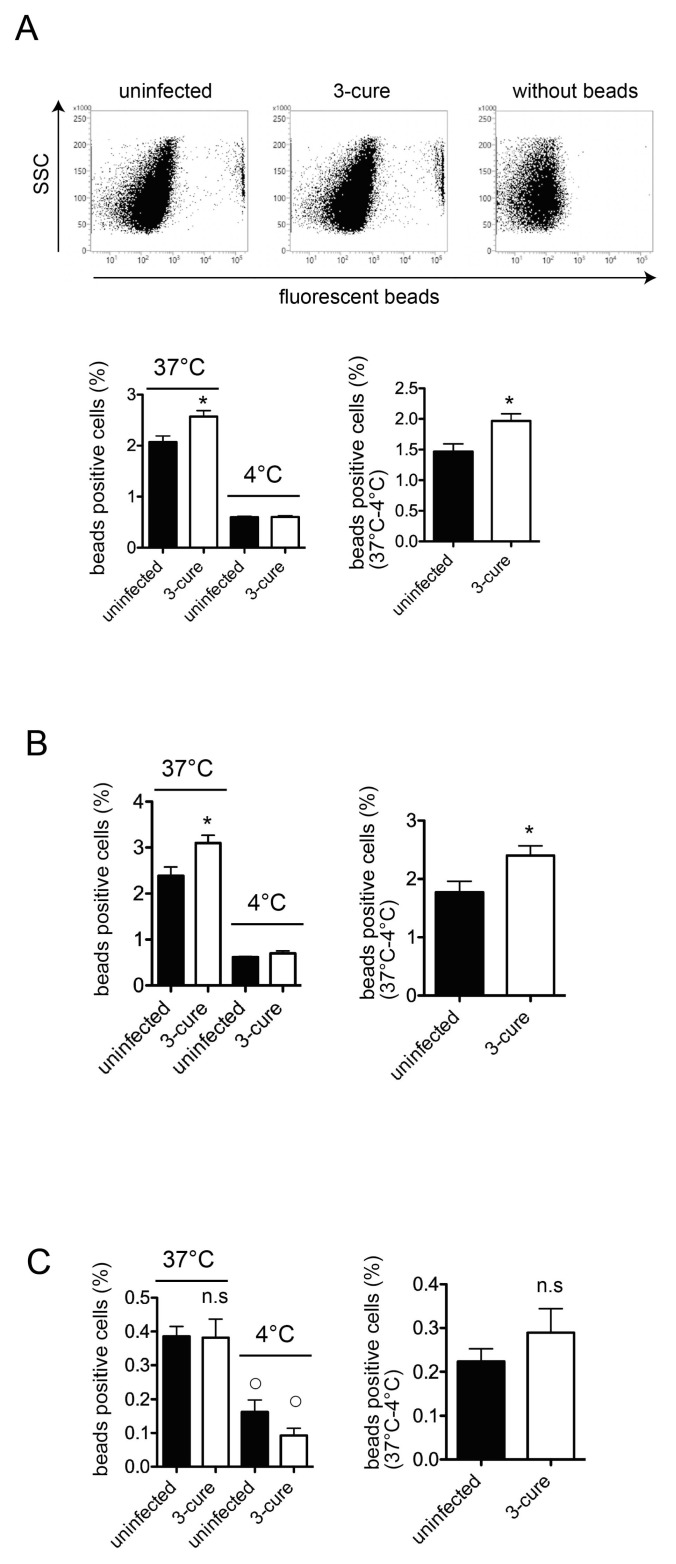
The capacity of in vitro phagocytosis of splenic CD8^−^CD11b^+^ cDC2 subset from 3-cure mice was increased compared to that from uninfected mice. The CD11c^+^ cDCs were purified from the spleens from uninfected or 3-cure mice, which were cultured with fluorescent beads for two hours at 37 °C or at 4 °C, and the proportions of fluorescent-positive cells in CD8^+^CD11b^−^ cDC1 and CD8^−^CD11b^+^ cDC2 subsets were determined. (**A**) The representative plots of CD11c^+^ gated cDCs cultured with fluorescent beads at 37 °C are shown. The percentage of fluorescent-positive cells of CD11c^+^ gated total DCs (n = 3 for each group) is demonstrated. (**B**) The percentage of fluorescent-positive cells of CD8^−^CD11b^+^ gated CD11c^+^DCs (n = 3 for each group). (**C**) The percentage of fluorescent-positive cells of CD8^+^CD11b^−^ gated CD11c^+^DCs (n = 4–5 for each group). The experiments were performed three times and representative data are shown. Asterisks denote that a significant difference was statistically observed between uninfected and 3-cure mice (*p* < 0.05). ○ in (**C**) shows a significant difference between 37 °C and 4 °C data in each mice group. n.s means not significant difference between uninfected and 3-cure mice.

Notably, the presence of fluorescent beads with cells at 4 °C indicated bead attachment to the cell surface, without internalization occurring [12]. The percentage of bead-positive cells within the total cells at 37 °C was significantly higher than at 4 °C (Figure 3A), indicating active phagocytosis at 37 °C in cDCs from both uninfected and 3-cure mice. As shown in Figure 3A, the capacity for phagocytosis of fluorescent beads at 37 °C was significantly increased in CD11c^+^ total cDCs prepared from the spleens of 3-cure mice compared to that from uninfected mice. 

Subsequently, we examined the capacity for phagocytosis in the cDC1 and cDC2 subsets, respectively. The total cDCs were divided into cDC1 and cDC2 subsets based on CD8 and CD11b expression. The capacity for phagocytosis of fluorescent beads at 37 °C was significantly increased in the CD11c^+^CD8^−^CD11b^+^ cDC2 subset prepared from the spleens of 3-cure mice compared to that from uninfected mice (Figure 3B). The capacity for in vitro phagocytosis of the CD11c^+^CD8^+^CD11b^−^ cDC1 subset was detected in both uninfected and 3-cure mice because the percentage of bead-positive cells at 37 °C was significantly higher than at 4 °C (Figure 3C). However, the capacity for in vitro phagocytosis of the CD11c^+^CD8^+^CD11b^−^ cDC1 subset was relatively lower than that of the CD11c^+^CD8^−^CD11b^+^ cDC2 subset in both uninfected and 3-cure mice (Figure 3C). There was no significant difference in the capacity for bead phagocytosis by the cDC1 subset between uninfected and 3-cure mice (Figure 3C).

## 4. Discussion

In this study, we investigated the phagocytic capacity of cDCs from 3-cure mice. Administering fluorescent beads into mice did not reveal enhanced phagocytosis in the spleens of 3-cure mice, but it did show that the percentages of cDCs within the fluorescent bead-positive cell population were significantly higher than in uninfected mice. Furthermore, our analysis of in vitro phagocytosis activity demonstrated that the cDC2 subset from 3-cure mice exhibited higher phagocytosis activity compared to that from uninfected mice. These results suggest that repeated infection–cure cycles modulated the phagocytosis activity of cDCs, particularly the cDC2 subset.

The phagocytic capacity of fluorescent beads can be correlated with the uptake of pRBCs. In a previous study, we demonstrated the uptake of pRBCs in the CD11b^+^F4/80^+^ population along with fluorescent beads [12]. The mechanism by which antigens from blood-stage parasites are delivered into CD11c^+^ cDCs in vivo has not been fully elucidated. However, as shown in Figure 2, the percentage of cDCs within bead-positive cells in the spleens was increased in 3-cure mice compared to uninfected mice, suggesting that more malarial antigens would be incorporated into cDCs in 3-cure mice. In contrast, the percentage of F4/80^+^ macrophages within bead-positive cells was decreased in 3-cure mice. F4/80^+^ macrophages have a lower capacity for antigen presentation to lymphocytes compared to cDCs. These observations suggest that more malarial antigens may be effectively utilized by cDCs to induce adaptive immunity against malaria infection in 3-cure mice than in uninfected mice, which may contribute to the potent anti-malarial effect observed in 3-cure mice.

This study also revealed that within the bead-positive cells in the in vivo phagocytosis assay, the percentage of the F4/80^+^ population was decreased, while that of the CD11c^+^F4/80^−^ population was increased (Figure 2). Several possibilities could explain this observation. First, the capacity for phagocytosis of cDCs may be increased in 3-cure mice. Therefore, we conducted an in vitro phagocytosis assay using purified cDCs. As shown in Figure 3, the capacity for phagocytosis of cDCs, especially the cDC2 subset, from 3-cure mice was increased compared to that from uninfected mice. However, the extent of the increase was not dramatic, and other possibilities should be considered. For example, the microstructure of the spleens may be altered in 3-cure mice. The microarchitecture of the spleen can change during malaria parasite infection [18]. The recovery process after repeated infection–cure cycles may alter the spleen’s microstructure, making blood-derived materials more accessible and easily incorporated into cDCs. Alternatively, the capacity for phagocytosis of F4/80^+^ macrophages from 3-cure mice may be decreased. The fluorescent beads that were not incorporated into F4/80^+^ macrophages could then be phagocytosed by cDCs.

In this study, we observed a significant increase in the phagocytic activity of the cDC2 subset from 3-cure mice compared to that of uninfected mice (Figure 3). However, the detailed mechanism underlying this phenomenon requires further elucidation. In this study, polystyrene fluorescent beads were employed for the phagocytosis assay. The scavenger receptor class A (SR-A, CD204) is known to be involved in the phagocytosis of polystyrene beads [8]. Notably, SR-A expression in the CD8^+^ cDC1 subset is lower than in the CD8^−^ cDC2 subset [19,20]. Previous studies have demonstrated that the cDC1 subset exhibited a lower capacity for bead phagocytosis compared to other cDC subsets [21]. These observations are consistent with the findings in this study, where the capacity for in vitro phagocytosis of beads in the cDC1 subset was lower than that in the cDC2 subset (Figure 3). Investigating the expression of receptors critically involved in enhanced phagocytosis in cDCs from 3-cure mice would be a valuable avenue for future research.

As demonstrated in a previous study, the proportion of cDC1 within the total cDC population was decreased in 3-cure mice [10]. These findings suggest that the role of the cDC2 subset in immune responses against antigens might be augmented in 3-cure mice compared to uninfected mice. The repetitive infection–cure cycle may modulate the balance between the cDC1 and cDC2 subsets to elicit immune responses. Previous studies have illustrated that the cDC1 subset is implicated in Th1 responses following *Plasmodium* parasite infection, contributing to the pathogenesis of ECM [7,22]. The shift towards the cDC2 subset over the cDC1 subset observed in 3-cure mice could potentially mitigate excessive Th1 responses and immunopathology. This concept aligns with “trained immunity”, where innate immune cells adapt their responses to subsequent challenges [23]. Increased transport of malarial antigens into the cDC2 subset through phagocytosis may result in a reduction in the activation of pathogenic CD8^+^ T cells. Furthermore, there may be an enhancement in the production of parasite-specific antibodies due to the preferential activation of B cells mediated by Th2-biased functions of the cDC2 subset, ultimately leading to the control of parasite growth. Recent research has introduced further classification of the cDC2 subset based on the expression of transcription factors such as T-bet or RORγt [24]. Therefore, it is crucial to investigate the proportions of these cDC2 subsets, specifically cDC2a and cDC2b, following *Plasmodium* infection–cure procedures.

## Data Availability

The data presented in this study are available from the corresponding author if requested reasonably.
